# Supplementary material for: Patterns of Intron Gain and Loss in Fungi
Source: PLoS Biol. 2004 Nov 30;2(12):e422. doi: 10.1371/journal.pbio.0020422 (PMC532390; doi:10.1371/journal.pbio.0020422)
Supplement: Table S1 — Also available at http://genes.mit.edu/NielsenEtAl/. (4.3 MB ZIP). [file pbio.0020422.st001.zip › NielsenEtAl/html/1312.html]

AN6521.1.NCU08898.1.MG04842.1.FG10949.1


```
 CLUSTAL W (1.82) Multiple Sequence Alignments - Introns Inserted


Sequence 1: MG04842.1	724 aa
Sequence 2: FG10949.1	776 aa
Sequence 3: NCU08898.1	784 aa
Sequence 4: AN6521.1	776 aa
Alignment Length: 800 aa
Number Identitical Residues: 421 aa
Alignment Score (without introns) 19853


MG04842.1 	-MLPA0RRSLALVATRGCR----RLPSLLPVVRRTQQVRP---LSSTPRLRQNAFHSQLQ
NCU08898.1	MIHPV0RRALAVAASRAPRQFLAAASRTTSVRSVRAAAASGSYYSTTSRRLQDAFPSQLE
FG10949.1 	-MVAL0RRAVALNAVAIAR----LQTRALTARTRSLALASSQYRAYKTSSRQHAFHSQLE
AN6521.1  	-----~MQSRLVSQSGLGR----RWAVLRCALSKTYQRRT---LTSTRRQFQDVFQSQLE
          	       ::  :      *      .    .        .    : .    *..* ***:

MG04842.1 	DTPSDLFSSFQTPP--SNPQTLTEKIVQRYSVGLAPGKKVKSGDYVTLSPHHCMT~HDNS
NCU08898.1	NLASSTLP--KVVP--QVPQTLTEKIVQRYSVGLAPGKKVKSGDYVTLQPHHCMT~HDNS
FG10949.1 	NTPTPSAFQYQKPPTVENPQTLVEKIAQQYAVGLAPGKKIKAGDYIALAPHHCMS~HDNT
AN6521.1  	DPTSAALFSALNSSK-AVPQTLTEKIVQKYSVGLPQGKFVKSGDYVTIQPHRCMT1HDNS
          	: .:    .    ..   ****.***.*:*:***. ** :*:***::: **:**: ***:

MG04842.1 	WPVALKFMSIGASKIHDNRQVV~MTLDHDVQNKSESNLKKYRQIEEFAKTHGVDFYPAGR
NCU08898.1	WPVAMKFMSIGASKIHDNRQVV~MTLDHDVQNKSEANLKKYRQIEEFANTHGVDFYPAGR
FG10949.1 	WPIAKKFLDIGASKIHNNRQVV1FTLDHDVQNKSEANLKKYSLIHEFAEKHGVIHYPAGR
AN6521.1  	WPCALKFMSIGASRLHNPDQIV~MTLDHDVQNKSDKNLKKYRQIEEFATQHGVEFYPAGR
          	** * **:.****::*:  *:* :**********: *****  *.***  *** .*****

MG04842.1 	GIGHQIMVEEG~YAWPGTVTVASDSHSNMYGGVGCLGTPMVRTDAASIWATGKT2WWQIP
NCU08898.1	GIGHQIMVEEG~YAWPGTVTVASDSHSNMYGGVGCLGTPMVRTDAASIWATGKT~WWQIP
FG10949.1 	GIGHQIMVEEG1YAWPGTVSVASDSHSNMYGGIGCLGTAVVRTDAASIWATGQT~WWQVP
AN6521.1  	GIGHQIMIEEG~FAWPGTLAVASDSHSNMYGGVGCLGTPIVRTDAASVWATGKT~WWQIP
          	*******:*** :*****::************:*****.:*******:****:* ***:*

MG04842.1 	PIAKVTLTGVLPPGVTGKDVIVALCGLFNNDEVLNHAIEFTGSEEQMRSIPVDDRLAIAN
NCU08898.1	PIAKVTFTGLLPPGVTGKDVIVALCGLFNNDDVLNHAIEFTGAEETMRSIPVDDRLTIAN
FG10949.1 	PVAKVTFTGILPPGVTGKDVIIALCGLFRDDQVLNHAVEFAGGE----SLPIDDRLTISN
AN6521.1  	PVAKVTFKGVLPPGVTGKDVIVALCGLFNKDDVLNHAIEFTGSEETMRSLSVDTRLTIAN
          	*:****:.*:***********:******..*:*****:**:*.*.   *:.:* **:*:*

MG04842.1 	MTTEWGALAGLFPIDSVLQGWLRGKATTSAMFGGG-------RDVFTHEKIEELIQNQLR
NCU08898.1	MTTEWGALSGLFPIDAVLASWMRAKATVTAMEHPELGD----KAKFTHAKIDALLENPLV
FG10949.1 	MTTEWGALAGVFPVDEMLISWYRGKATTNAMFGGSS------KDRINHKRVDELEQNRLE
AN6521.1  	MTTEWGALSGLFPIDSVLKGWLRGKATTAAMGLADGPFKTRAAERFTHPLLEQLFENPLT
          	********:*:**:* :* .* *.***. **       .: :   :.*  :: * :* * 

MG04842.1 	ADPGAKYAKSLYLNLSTLAPFVAGPNSVKVATPLKDLEAKDIKVNKAYLVSCTNSRASDI
NCU08898.1	ADPGATYAKQLYLNLSTLSPFVAGPNSVKIATPLKDLEAQNLKLDKAYLVSCTNSRASDI
FG10949.1 	ADPNAKYAKELYLNLSTLSPIVAGPNSVKIATPLKNLEAEDIPVNKAYLVSCTNSRASDI
AN6521.1  	ADKGAKYAKELFLDLSSLSPYVSGPNSVKVATPLKELEAQNIKVDKAYLVSCTNSRASDI
          	** .*.***.*:*:**:*:* *:******:*****:***::: ::***************

MG04842.1 	AAAAKVFKEASKDGKPAV--IAPGVKFYIGAASTLEQAAAEEAGDWKVLLDAGAQALPAG
NCU08898.1	AAAARVFKDAAADNNGVIPKVAPGVKFYIAAASLLEQKQAEDSGDWQVLLDAGAEPLPSG
FG10949.1 	AAAARVFREAAKENKDVK--IAPGVNFYIAAASLPEQEIAEEAGDWQVLRDAGAQVLPAG
AN6521.1  	AAAAKVFKEAAEKNGGKIPKIADGVKFYIAAASIPEQLAAEGNGDWQTLLEAGATQLPAG
          	****:**::*: ..    ..:* **:***.***  **  **  ***:.* :***  **:*

MG04842.1 	CGPCIGL~GTGLLEPGEVGISASNRNFK~GRMGSTDAKAYLASPEVVAASALKGKIAGPG
NCU08898.1	CGPCIGL~GTGLLEPGEIGISASNRNFK~GRMGSTDAKAYLASPEVVAASALKGKIAGPG
FG10949.1 	CGPCIGL0GTGLLEPGEVGISASNRNFK0GRMGSTSAKAYLASPEIVAASALKGKIAGPG
AN6521.1  	CGPCIGM~GQGLLEPGEVGISASNRNFK~GRMGSTEAKAYLGSPEVVAASALSGKLSGPG
          	******: * *******:********** ******.*****.***:******.**::***

MG04842.1 	WYEKPEGVEKVIIGEGNGDAVHDKAVSIEEALAKLLGEAESMISDAEQNLG--GSSQQAE
NCU08898.1	WYQKPEGVEKVIIGEGNGVVEQDKAMSIEDALDKIIAEAESLIANAEAGLTPESTSSSSS
FG10949.1 	WYQKPEGVEKVIIGEGSGDYVADKALSIEDALDKLINEADALIAAAETSEG--AKEQAAS
AN6521.1  	WYQTPEGWTEVIRGEGDGIREEDRMLTNEEALEKIIGQLDDLVADGEKRFA------SET
          	**:.***  :** ***.*    *: :: *:** *:: : : ::: .*             

MG04842.1 	S---AEEETLTEILPGFPERVEGEIVFCDADNINTDGIYPG~KYTYQDNVSTEKMAEVCM
NCU08898.1	SSSSSEEESLTEILPGFPEAISGEIIFCDADNINTDGIYPG~KYTYQDNITPAKMAEVCM
FG10949.1 	PTAAEGEESLTEILPGFPEKVEGEIVFCDSDNINTDGIYPG1KYTYQDNVSVEKMAEVCM
AN6521.1  	PAVEESEQGLTEIYPGFPERVSGELVFCDADNVNTDGIYPG~KYTYQDDVPPETMARVCM
          	.:    *: **** ***** :.**::***:**:******** ******::.  .**.***

MG04842.1 	ENYDKEFGKVAREGDVLVAGFNFGCGSSREQAATAILAKRIPLVVAGSFGNIFSRNSINN
NCU08898.1	ENYDASFGSIARAGDILVTGFNFGCGSSREQAATAILAKQIPLVVSGSFGNIFSRNSINN
FG10949.1 	ENYDTEFGKFAKAGDILVTGFNFGCGSSREQAATALLAKKIPLVVSGSFGNIFSRNSINN
AN6521.1  	ENYDPEFSTTAKEGDILVSGFNFGCGSSREQAATAILAKKIPLVVSGSFGNIFSRNSINN
          	**** .*.. *: **:**:****************:***:*****:**************

MG04842.1 	ALMGVEVPRLVERLRETFGGKEQGEENKALTRRPGL------------------------
NCU08898.1	ALMGVEVPKLVQRLREEFGD-------KVATRRTGWKLTWDVRRSKVVVEE-ASGKKWEQ
FG10949.1 	ALMGVEVPRLVERLRETYKN----DTEKPLTRRTGWKLVWDVRRSKVIVTE-KDGSSWEQ
AN6521.1  	ALMGLEVPRLVNRLRETFGS-----GDKVLTRRTGWTLTWDVRKSQIEVQEGPGGPKWTH
          	****:***:**:**** : .      .*  ***.* .   .   :.    .. .. .   

MG04842.1 	----------------------------
NCU08898.1	KVGELPPNVQEIIARGGLEKWVKSQIEA
FG10949.1 	KVGELPANVQEIIARGGLEKWVKAKIDA
AN6521.1  	KVGELPPNVQEIIAKGGLEKWVKNAIGA
          	. .. ... ..  : .. ..  .    :
```
